# Constant regulation for stable CD8 T‐cell functional avidity and its possible implications for cancer immunotherapy

**DOI:** 10.1002/eji.202049016

**Published:** 2021-03-30

**Authors:** Connie B. Gilfillan, Michael Hebeisen, Nathalie Rufer, Daniel E. Speiser

**Affiliations:** ^1^ Department of Oncology University Hospital and University of Lausanne Lausanne Switzerland

**Keywords:** avidity regulation, CD8 T cells, coreceptors, functional avidity, TCR affinity

## Abstract

The functional avidity (FA) of cytotoxic CD8 T cells impacts strongly on their functional capabilities and correlates with protection from infection and cancer. FA depends on TCR affinity, downstream signaling strength, and TCR affinity‐independent parameters of the immune synapse, such as costimulatory and inhibitory receptors. The functional impact of coreceptors on FA remains to be fully elucidated. Despite its importance, FA is infrequently assessed and incompletely understood. There is currently no consensus as to whether FA can be enhanced by optimized vaccine dose or boosting schedule. Recent findings suggest that FA is remarkably stable in vivo, possibly due to continued signaling modulation of critical receptors in the immune synapse. In this review, we provide an overview of the current knowledge and hypothesize that in vivo, codominant T cells constantly “equalize” their FA for similar function. We present a new model of constant FA regulation, and discuss practical implications for T‐cell‐based cancer immunotherapy.

## Graphical abstract

The functional avidity (FA) of CD8 T cells is stably maintained, and similar despite different TCR affinities of the codominant clonotypes. We hypothesize that continued adjustment of the net coreceptor signal strength (i) compensates for the differences in TCR affinity and (ii) acts to keep FA stable despite changing T‐cell activation stages.

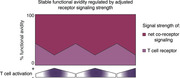

## 1 Introduction

For approximately 30 years, it has been known that successful cellular immune responses depend on robust interactions of T cells with cognate antigen‐bearing cells [1]. The affinity of the TCR to peptide‐MHC (pMHC), hereafter “TCR affinity,” is a central parameter determining the strength of the T‐cell interaction [2]. TCR affinity refers to the biophysical strength by which a TCR binds to a given pMHC complex (Box 1). However, T‐cell interactions involve much more than TCR‐pMHC. The immune synapse is complex, engaging multiple functionally relevant coreceptors and ligands on the membranes of interacting cells [3]. Therefore, cellular assays are required to evaluate the overall functional potency of CD8 T cells resulting from those interactions. The most frequently used assays measure the efficacy of target cell killing (cytotoxicity) or production of cytokines (typically IFN‐γ) at distinct peptide densities on target/stimulator cells. These assays determine the T‐cell's functional avidity (FA), which is depicted as the peptide concentration at half‐maximal T‐cell activity (EC_50_) [4].

Generating and boosting tumor antigen‐specific T‐cell responses has become an important aim for improving cancer immunotherapy. Researchers and developers often ask the question: how can we induce high FA T‐cell responses? For antibody‐inducing vaccines, well‐known vaccination guidelines exist. These are based on the fact that B cells undergo antibody affinity maturation that can be strategically maneuvered by the choice of antigen, dose, and the timing of short‐term boost vaccinations [5, 6]. Consequently, vaccine antigens and doses must be carefully optimized, and time intervals for booster vaccinations must be at least 4 weeks to assure the generation of high affinity antibodies. B cells producing high affinity antibodies are more selectively boosted after longer vaccination intervals [7]. Unlike antibodies, which act as freely circulating molecules, proper T‐cell function continuously depends on the highly complex regulation of eukaryotic cell‐cell interactions involving many receptors and ligands and their functions. Furthermore, the TCR of any given T cell does not change as in contrast to B‐cell receptors, TCRs do not undergo somatic hypermutation and affinity maturation. TCR‐pMHC affinities are relatively weak, with *K_D_* values that may range between 100 and 1 μM, in contrast to antibodies that may reach very high affinities (*K_D_* values 100 to 1 pM) [2]. Finally, it remains unclear whether T cells can undergo avidity maturation during the primary immune response [8]. If primary avidity maturation was established and occurred in epitope‐specific CD8 T‐cell responses, then vaccines should/could be optimized for this phenomenon to occur. However, this has not been the case and the advancement of improving T‐cell vaccines for increased FA is still an ongoing process.

**BOX 1: Definitions of TCR affinity and functional avidity (FA)**

**TCR affinity**: Simplified term used for the TCR‐peptide‐MHC affinity, as determined with recombinant proteins, using surface plasmon resonance (SPR).

**Functional avidity (FA)**: The peptide concentration mediating half‐maximal T‐cell responses (EC_50_) in functional assays (e.g. cytokine production or cytotoxicity). FA depends on the TCR affinity as well as the TCR affinity‐independent interactions of coreceptors and ligands in the immune synapse, and their downstream signaling.

## 2 CD8 T‐cell correlates of protection

Before discussing FA, we provide a brief summary of T‐cell properties that correlate with protection from intracellular pathogens and tumors. First, sufficient activation of T cells is required to produce a functional response, through antigen stimulation via the TCR and the involvement of coreceptors. This is triggered by ligands on antigen presenting cells (APCs) and stimulatory cytokines, particularly IL‐12 [9, 10]. Successful activation results in expansion, differentiation, homing, and effector functions of T cells. Polyfunctional T cells are able to produce multiple cytokines, such as IL‐2, TNF‐α, and IFN‐γ, which can correlate with protection in some circumstances, such as HIV or CMV infection [11, 12]. Besides producing cytokines, CD8 T cells are cytolytic, which may be needed for immune defense; for example, from murine LCMV infection [13].

The magnitude of the CD8 T‐cell response is frequently linked to protection from viral infection, as shown in humans and mice [14, 15, 16]. In addition, highly polyclonal T cells specific for multiple different epitopes have been shown to be more effective than oligoclonal responses in humans [17, 18]. Among T cells specific for a single epitope, a large number of clonotypes is beneficial as it may compensate for losing some clonotypes during the immune response [19]. Similar principles may apply for antitumor responses in mice [20]. As detailed below, it seems important to avoid major selective outgrowth of a few clones, at the expense of others.

The stability of the peptide binding to MHC correlates with protection, and the pMHC stability is a good predictor of CD8 T‐cell immunogenicity [21]. The properties of antigens themselves likewise is relevant for successful T‐cell responses. The amount of presented peptide may impact on the number, and potentially the quality of responding human T cells [22, 23].

A major factor is the strength by which T cells interact with cognate antigen‐bearing cells, correlating with protection from disease, whereby the TCR affinity plays a central role (reviewed in Refs. [24, 25]). During priming, a large number of TCRs (clonotypes) are recruited, depending on the TCR affinity determined by the pMHC‐binding regions of the TCR‐αβ chains. The CDR3 regions are important for peptide binding that rely on CDR3 length and sequence, as demonstrated for influenza virus‐specific TCRs [26, 27, 28]. The recruited TCR repertoires may be highly diverse, depending on the type of antigens (e.g. viral, bacterial), the dynamics and duration of the pathogen presence, and the patient's HLAs and age. Furthermore, TCRs are often cross‐reactive to different pMHC complexes [29, 30]. Consequently, encounters with previous or persistent antigens influence the T‐cell responses to the antigens of a given disease. The knowledge on TCR sequences, structures, functions, and dynamics is already enormous and rapidly increasing (reviewed in Refs. [19, 31–33]).

## 3 Measuring the strength of T‐cell interaction

FA assays are widely used because they represent a standard method with which different immune responses and different molecular techniques can be compared and validated. However, FA assays are not always technically feasible (Box 2) and of limited reproducibility, particularly if done at differing T‐cell activation stages. Therefore, scientists make increasing use of sophisticated molecular tools to study the strength of T‐cell interactions, focusing on individual parts of the immune synapse and downstream signaling. Such techniques are 2D‐measurements, analysis of slip and catch bonds, high‐resolution imaging, force analysis with DNA tension probes or optical tweezers, affinity studies with recombinantly expressed TCR‐pMHC molecules, and off‐rate measurements on living T cells [34, 35, 36, 37, 38].

**BOX 2: Method for determining the functional avidity (FA)**

FA assays determine the peptide concentration‐dependence of T‐cell activity (killing or cytokine production). A mathematically correct calculation of the FA (EC_50_) is only possible from peptide titration data describing a complete sigmoid curve (e.g. black curve). In some studies, the FA is determined using incomplete titration curves, that is, curves lacking the low or the high plateau (e.g. grey curve), yet such curves do not permit the calculation of EC_50_. Incomplete curves may be obtained from T cells that display only low maximal activity (killing or cytokine expression), which is often the case for T cells that are only weakly activated, and T cells with low‐affinity TCRs.

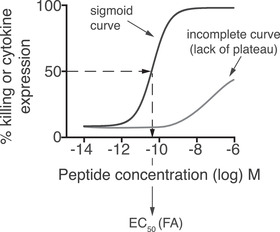

The TCR‐pMHC affinity (*K_D_*), as well as its dissociation (*k*_off_) and association (*k*_on_) rates can be measured at the molecular level by surface plasmon resonance (SPR) [39, 40, 41]. More recent advancements in tetramer technology led to the development of reversibly binding two‐color fluorescent pMHC streptamers and NTAmers (pMHC tetramers with Ni^2+^‐nitrilotriacetic acid‐His‐tag). Once bound to TCRs, these reagents can be cleaved to pMHC monomers that enable the calculation of monomeric TCR‐pMHC dissociation rates directly on living T cells by flow cytometry [42, 43, 44]. This technology provides the opportunity to distinguish stronger and weaker antigen‐binding TCR clonotypes without the need to produce recombinant TCR protein required for SPR measurements.

One of the great challenges is to combine biophysical approaches with readouts of cellular function [45, 46]. While the latter is done with FA assays, they only evaluate the T‐cell's functional dependence on pMHC density, and do not provide insights into molecular interactions within the synapse and downstream signaling. This also makes them susceptible to variations depending upon the context at which the EC_50_ value is being measured, for example, APC used, cytokines present in culture, etc. With novel laboratory techniques, it may become possible to improve measurements that allow for integration of the overall physical interactions, consequent signaling events, and cellular function.

Investigations are mostly done with T‐cell clones or TCR transgenic cells. They provide enough cells for experimentation and limit the numbers of involved TCRs, making it more feasible to precisely determine structure/function relationships. Additional efforts are required to link data from this type of “precision immunology” with the observations in patients and mice with their natural polyclonal T‐cell repertoires. However, high numbers of different TCRs pose enormous challenges, in addition to the dynamic functional changes. Most novel laboratory technologies for the assessment of the strength of T‐cell interactions need further improvement such that they can be applied to natural polyclonal T‐cell responses. Therefore, FA assays remain an important standard.

## 4 The functional avidity of CD8 T cells

The FA not only depends on TCR affinity but also on the coreceptors and their ligands in the immune synapse, along with the strength of their downstream signaling [47]. The overall intensity of interaction and function is reflected in the FA (EC_50_; Box 1), highlighting that FA could be considered as the most comprehensive correlate of protection. In vitro experiments showed that murine CD8 T cells with high FA provide better protection against viral infections compared to those with low FA [48, 49]. Murine T cells with high FA may be able to lyse infected cells earlier by recognizing lower antigen density and killing more rapidly [49]. For tumor antigens, it was found that after in vitro generation of CD8 T cells with high and low FA, it was the high FA cells that conferred better in vitro and in vivo antitumor activity in mice [50]. There are many murine and human studies showing that CD8 T cells with higher FA correlate with improved protection from viruses and tumors [51, 52, 53, 54, 55].

Although TCR affinity and FA have proven to be of central importance, they are not often evaluated in vaccination and immunotherapy studies, where they could potentially be very informative. Moreover, the field has been held back by a lack of systematic evaluation and method standardization. It is known that the in vitro measured FA can vary depending on the T‐cell's activation stage [46] and is, therefore, influenced by the dynamic fluctuations occurring in the immune synapse. These fluctuations can modulate FA through changes in the involvement of signaling of TCR and CD8, adhesion molecules, as well as activating and inhibitory coreceptors [44]. Furthermore, multiple supramolecular mechanisms are involved including oligomerization [56], membrane organization [57], and lipid rafts [58]. It is still only partially understood which mechanisms play major roles in FA regulation.

The fact that FA can be modulated independently to TCR affinity is well established [59]. This has been named the TCR affinity‐independent regulation, as observed in human HIV‐specific CD8 T cells [60], as well as monoclonal LCMV‐specific T cells in mice [61]. Avidity modulation via coreceptors and their signaling may be supported by epigenetic programming [62]. For cytokine expression, there is indeed evidence for epigenetic control (DNA demethylation/histone acetylation), as reported in a study investigating mechanisms that explain why murine memory CD8 T cells have enhanced cytokine production over primary CD8 T cells [63]. However, in this example, there was no FA difference.

Analyzing human tumor antigen‐specific T cell clones in vitro, it has been shown that FA correlated with the expression levels of several coreceptors, such as CD8‐αβ, CD28, VLA‐1, VLA‐4, CD137, CD5, LAG‐3, and TIGIT [44], similar to the TCR‐αβ expression level. However, the correlation between the expression level of any receptor and FA should not lead to the conclusion that this receptor plays a role in FA regulation. For example, deletion of CD28 did not affect the FA of CD8 T cells in immunized mice [64] even though CD28 plays a key role in TCR signaling [65] and supports the generation of a functional synapse, as reviewed in Ref. [66].

Examples of receptors that have been shown to modulate the avidity of CD8 T cells are Ly108 and 2B4, two members of the signaling lymphocytic activation molecule (SLAM) coreceptor family. They dynamically influence the localization and activity of Src kinases in the synapse. This became evident in studies of patients with X‐linked lymphoproliferative syndrome, caused by mutations of the SAP adaptor protein that links SLAM family receptors to downstream signaling [67].

CD8‐αβ is probably the best studied coreceptor regarding its functional impact on the strength of T‐cell interactions. CD8‐αβ binds to MHC class I, and therefore, also contributes to the binding of pMHC tetramers and monomers. From a naïve to an antigen‐experienced T cell, there is a change in the membrane distribution of TCR and CD8 molecules, with close proximity of the two, enhancing the sensitivity to antigen [68, 69]. Importantly, CD8 binding also facilitates Lck delivery to the TCR‐CD3 complex and, thus, its downstream signaling [70].

The level of programmed cell death protein‐1 (PD‐1) expression does not appear to correlate with FA [44], a finding that however does not rule out that PD‐1 may modulate FA. Interestingly, anti‐PD‐1 checkpoint blockade therapy in melanoma patients was found to promote high‐affinity T‐cell clonotypes, and in vitro blockade of PD‐1 on human Melan‐A‐specific T cells induced amplification of those with a TCR repertoire with higher FA [71].

The mechanistic characterization of TCR affinity‐independent FA regulation is facilitated when using monoclonal T‐cell populations, due to sharing of a single TCR type [8, 61, 72]. Factors, such as timing and degree of T‐cell activation, coreceptor participation and resulting signaling strength may all have the potential to affect the FA of the T‐cell population. To investigate the specific influence of each (co‐)receptor molecule in the synapse, experiments need to be designed with a series of single KO of coreceptors (e.g. CD5, PD‐1). It would require methodical assessment of the impact on the T‐cells’ binding strength, signaling, and consequent cellular functions. For most synapse molecules this is yet to be done. Because of (potential) roles in thymic selection and other cell types, such experiments should be done with conditional and cell‐specific KO mouse models. Additional studies are necessary to determine changes in receptor conformation and clustering in the immune synapse, representing considerable challenges for elucidating the implied mechanisms. For example, enriched TCR oligomerization was found to contribute to the increased sensitivity of antigen‐experienced T cells [56].

For the time being, the field may at least build on the observation that FA assays should be performed with T cells at a similar and standardized activation stage. This will enable meaningful comparisons and conclusions on avidity differences and potential avidity maturation. Many questions remain about the involvement of TCR affinity‐independent factors, with more elucidation requiring further experiments in closely controlled conditions. There may be strong differences of FA observed in vivo as opposed to in vitro [8]; therefore, one has to be careful in making assumptions of one to the other considering the large differences in cell involvement, inflammation, controlled activation, etc. Nevertheless, both in vivo and in vitro analyzes offer their own advantages and disadvantages, and both are important to help expound the mechanisms involved in FA regulation.

## 5 Each epitope appears to have its own rules

There is sufficient evidence to suggest that for the FA‐associated parameters discussed above (such as TCR affinity, pMHC stability, antigen density, polyfunctionality, killing, protection from disease) the rules are specific for a given epitope and may not necessarily apply across different epitopes. A seminal article from Gallimore et al. looked at the three codominant epitopes involved in the CD8 T‐cell response to LCMV infection in C57BL/6 mice. The T cells specific for the NP396 epitope had the strongest binding to pMHC and the highest FA, conferring the best protection. The least protective were the T cells specific for the GP33 epitope, despite the highest T‐cell frequency and the largest peptide density on infected cells. The T‐cells specific for the third epitope, GP276, showed intermediate pMHC binding, FA, and protective power, while having the lowest T‐cell frequency and the lowest TCR diversity [73]. Such observations suggest that the FA‐associated parameters may be compared between the T cells specific for a given epitope, but not across different epitope specificities, even within a single infection in which several different epitope‐specific responses contribute to protection. Another murine study found that the T cells specific for four different influenza epitopes had differences in their functionality, as measured by IFN‐γ and TNF‐α production [74]. More recent studies in mice with mousepox and vaccinia virus, respectively, showed that subdominant epitopes can also elicit protective immunity, not only the immunodominant ones [75, 76]. Moreover, there are further parameters that influence epitope‐specific responses, such as T‐cell precursor frequency [77] and T‐cell competition [78, 79]. It seems likely that focusing immunotherapies solely on the most immunodominant epitopes may not lead to optimal protection from disease. Together, these results highlight the importance of studying each epitope‐specific response separately, as there could be a coexistence of different T‐cell populations specific for different epitopes that each bear their own rules, yet together may confer protection from disease.

## 6 Generation of a primary CD8 T‐cell response

**Figure 1:**
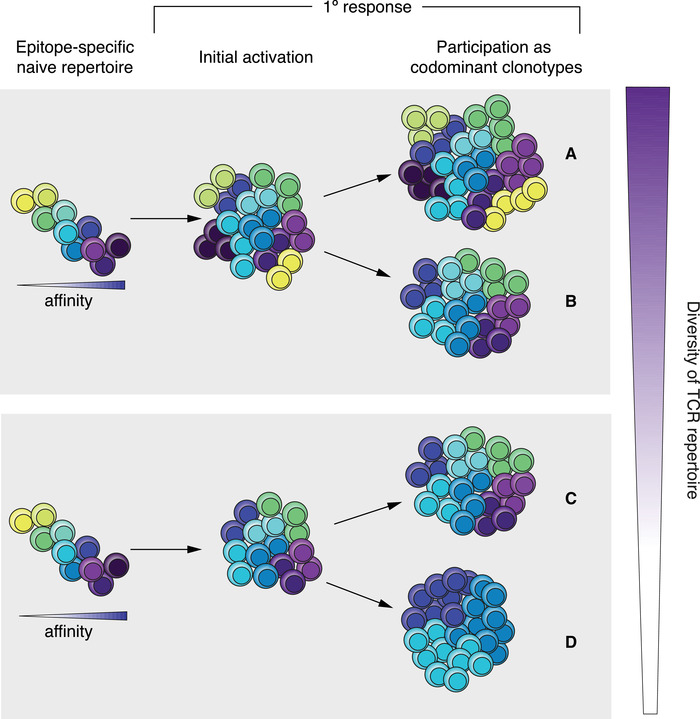
Four scenarios of how CD8 T‐cell clonotypes may evolve over time in a primary response to a given epitope. Any T cell may participate to an immune response if its TCR is sufficiently capable of recognizing a given epitope (left). During priming, either all those T cells are activated (top) (A, B) or only the ones with more favorable TCR affinities (bottom) (C, D). Those that will become codominant clonotypes (i.e. the T cells making up the majority of cells in a given epitope‐specific response) in the fully established primary immune response (right) may be all of the initially activated clonotypes (A) or only a selection based on optimal TCR affinity (B, D). The TCRs with the different affinities are shown in different colors, from yellow (lowest affinity) to dark purple (highest affinity).

T cells are selected to participate during an immune response depending on the efficacy of their TCRs to recognize antigen. However, it is still not fully understood how the CD8 T‐cell repertoire is established and eventually modified over the course of the primary immune response. During priming, one may consider different scenarios for the establishment of an antigen‐specific polyclonal T‐cell population. There could be activation of T cells with a wide range of TCR affinities, from low to high TCR affinity, without initial narrowing of the repertoire [28]. This could establish codominant clonotypes consisting of the entire range of TCRs that were initially activated (Fig. 1A), or there could be an early loss of those with the lowest affinity and eventually also the highest affinity (Fig. 1B) [80]. Another alternative is the narrowing of clonotypes already during initial activation (Fig. 1C) [81]. Finally, there could be further narrowing of the repertoire down to relatively few clonotypes that partake in the antigen‐specific response (Fig. 1D). In contrast to comparisons of primary with secondary immune responses [82, 83], the dynamics suggested here of clonal selection during the primary response are difficult to assess experimentally. Therefore, it remains unknown which of these four scenarios (Fig. 1) is actually correct.

**Figure 2:**
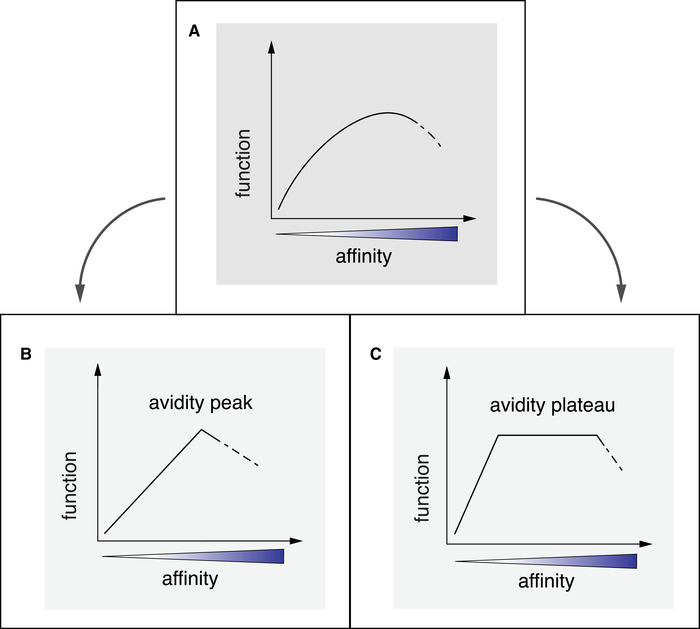
Three possibilities of clonal distribution of an epitope‐specific CD8 T‐cell population. The TCR affinity of clonal T cells (X axis) is shown in relation to their functional capabilities (Y axis; functional avidity, proliferation, cytokine production, killing; all these capabilities usually correlate with each other). T‐cell functions correlate with TCR affinity up to a certain level, above which the function gets weaker due to unfavorably strong TCR interactions and overcompensatory negative signaling. Therefore, TCR affinities may be distributed along a bell‐shaped curve (A). One may expect a very high degree of selective advantage for those T‐cell clonotype(s) with ideal affinity TCRs mediating optimal functionality and, therefore, becoming highly dominant (B). However, this does not seem to be the case in the majority of CD8 T‐cell responses. Therefore, there may exist a range of TCRs conferring similar functionality (through compensatory amplification and/or inhibition by coreceptors in the immune synapse) despite different TCR affinities, corresponding to a plateau composed of T cells with TCRs within a favorable range of affinities mediating equal function (C).

It may be assumed that the recruitment of cells will occur according to their TCRs, with priority given to those mediating best function, followed by close‐to‐best function, and low priority to cells with TCRs mediating low function (Fig. 2A). This is expected as the TCRs involved in a given epitope‐specific T‐cell response are enriched for mediating optimal function, but that TCRs with lower and higher affinities are also involved. Possibly, this scenario may not be durable as selection mechanisms could progressively give disproportional advantage to only one or a very low number of TCRs mediating the best function, such that the response will become dominated by very few T‐cell clonotypes (Fig. 2B). Alternatively, a larger number of clonotypes may participate codominantly in the response, with a range of TCR affinities that all mediate similar optimal avidity and function [84]. Accordingly, a “plateau” may be postulated in which T cells bear TCRs with a range of affinities that mediate equal and optimal function (Fig. 2C). Indeed, this is what was found in several studies [85, 86]. Such a scenario seems preferable, as it may support the participation of larger numbers of clonotypes and, thus, avoid the outgrowth of only very few highly dominant clones. For T cells specific for the human tumor antigen A2/NY‐ESO‐1, optimal and equal function was shown to be mediated by TCR affinities (*K_D_*) within the range of 5 to 1 μM [87].

As mentioned, it is reasonable to assume that those T cells with too low‐affinity and too high‐affinity TCRs will not significantly participate in a primary immune response. With “too low” and “too high,” we mean affinities that mediate clearly reduced functionality. Cells with very low‐affinity TCRs are poorly functional [88, 89] and may, therefore, not significantly contribute to the response. Conversely, very high affinity cells can display decreased functionality due to increased negative regulation. Indeed, there is an upper TCR affinity limit, above which there is no improvement [87, 90]. In addition, cells with high TCR affinity may undergo activation‐induced cell death, associated with decreased expression of Bcl‐2 [91]. Therefore, affinity thresholds likely exist, impacting T‐cell function both at the low and the high end of TCR affinity for pMHC. We hypothesize that the T cells within the above‐mentioned TCR affinity range of optimal function [87] may be the ones that dominate the primary response.

## 7 FA in the primary immune response and effects of inflammation

It remains unclear whether T cells undergo FA maturation in the primary response, either induced naturally or through vaccine priming and short‐term boosting. It is possible that avidity maturation is largely context‐specific (e.g. specific to certain viral infections). While several studies showed avidity maturation [61, 92], the majority report maturation of maximal T‐cell function, rather than maturation of FA (EC_50_). This reflects the current status of knowledge, namely that T cells may progressively increase their function but not their FA. The distinction between these two functional criteria is also important for rigorous technique and interpretation of FA assays (Box 2).

Inflammatory triggers may influence FA. In fact, most of what is known about potential avidity maturation in vivo has been observed only in murine models of infection. *Listeria Monocytogenes* infection was shown to drive selective expansion of higher affinity T cells [92], as well as improve effector functions [93]. Others have shown that inflammatory cytokines, such as IL‐12 and type 1 IFNs, directly regulated the antigen sensitivity of CD8 T cells, in a way that was independent of clonal selection [94]. Moreover, inflammation was important for controlling specific functional aspects of the T‐cell response. Studies using heterologous boosting in mice exploited this effect by changing the vaccine formulation from the prime to enhance inflammation and improve FA [95, 96]. What these results suggest, is that the inflammatory response driven by infection may impact the function of T cells and eventually also their FA.

## 8 Secondary immune response and chronic/latent infection

There is a clear evidence for clonotype selection in secondary infection [31, 97]. One possible reason may be the skewing of the TCR repertoire in the memory stage. Significantly reduced cross‐reactivity was found in the secondary, as compared to the primary response to LCMV in mice, suggesting narrowing of the TCR pool [98]. Several studies demonstrated selection of murine CD4 and CD8 clonotypes with increased TCR affinity and higher FA during the secondary response [52, 92]. It is clear that modified clonal dominance in the secondary response can impact the FA, by selecting for clones with higher affinity TCRs. For further details regarding memory/secondary responses, we refer to comprehensive reviews [31, 99].

Besides primary and secondary responses, a third condition is represented by the continuous long‐term presence of antigen, for instance, in cancer patients and in chronic or latent infections. This condition may elicit long‐lasting T‐cell responses, enabling investigations of long‐term clonotype evolution. Interestingly, many human studies observed remarkable clonotypic stability over long periods of time. In melanoma patients, the clonality of CD8 T cells specific for the tumor antigens NY‐ESO‐1 and Melan‐A was found to be stable over many years [100, 101, 102]. Similarly, influenza virus‐ and EBV‐specific clonotypes appear to persist long term [26, 27, 103, 104, 105]. In contrast, loss of some CD8 clonotypes and, thus, possible changes of the overall TCR affinity may occur in T cells specific for HIV or CMV. This is presumably related to the exceptionally strong and/or long‐term stimulation exerted by these viruses [106, 107]. Stability of some but not all clonotypes was found in exhausted LCMV‐specific CD8 T cells [108]. Together, some of the clonotypes that are very strongly and/or long‐term antigen exposed may disappear, whereas most of the remaining types of immune responses show clonotypic stability and stable FA.

## 9 A new model for explaining FA stability in vivo

**Figure 3:**
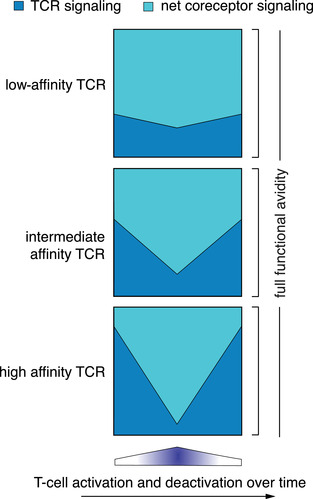
New model for similar and stable functional avidity (FA), independent of TCR affinity and T‐cell activation stage. Examples of three T cells that express TCRs with three different affinities (low, intermediate, high), together participating in an epitope‐specific primary response. “Net coreceptor signaling” (light blue) refers to the net signaling mediated via inhibitory and activating coreceptors. The degree of this net coreceptor signaling may be complementary to the TCR signaling (TCR affinity‐dependent; dark blue), together mediating similar and stable functional avidity. Those T cells with lower affinity TCRs may have a higher assistance of activating coreceptor signaling, and reduced inhibitory signaling, resulting in similar FA compared to the higher affinity TCRs. T cells with intermediate affinity TCRs may have intermediate levels of both TCR and net coreceptor signaling. Over time, when T‐cell activation first increases and subsequently decreases again after the peak of the response, TCR signaling conversely decreases and increases again, due to TCR down‐ and upregulation. In parallel, the net coreceptor signaling may increase and decrease again, compensating the changes in TCR signaling depending on the T‐cell's activation stage.

Recently, we analyzed the FA of murine CD8 T cells in response to homologous peptide vaccinations and compared 2‐ with 4‐week prime/boost intervals or low with high antigen density vaccines. Interestingly, we found similar FA irrespective of the different vaccination strategies. Moreover, FA was surprisingly stable over time in vivo [8]. There may be well‐founded reasons why T cells could profit from keeping FA stable and similar among the cells that participate in the response. T‐cell recognition underlies fundamentally different principles as compared to antibody binding. Low‐dominant antibodies may not necessarily be outcompeted by a few highly dominant ones. Antibodies are isolated soluble molecules, of which high‐ and low‐affinity versions may coexist and function in parallel. The situation for T cells may be more competitive than for soluble molecules. Cells must strive for numerous factors, such as space, access to critical areas, interaction partners, and nutrients for growth. If an optimal TCR gives the T cell a selective proliferation advantage, there may be an elevated risk that these T cells outcompete those with less favorable TCRs. Consequently, only a few high affinity clonotypes would prevail, which would weaken the potential of T‐cell responses generated out of large numbers of naïve T cells. Therefore, the system may function best when the T cells that participate in an epitope‐specific T‐cell response “equalize” their FA for similar function. This may apply to all T cells expressing TCRs within a favorable range of affinities mediating best and similar function. Therefore, we propose a new model (Fig. 3): T cells with high affinity TCRs will have stronger TCR signaling, but less net signaling via the coreceptors (costimulators minus coinhibitors). In contrast, T cells with lower affinity TCRs will have weaker TCR signaling, but stronger net signaling via the coreceptors. Moreover, the stage of T‐cell activation will change over time, with concordant variations of the TCR expression level, lowering when highly activated and increasing again over time.

FA tuning has been described previously: flexibility in the selection and development of T cells in the thymus was found to be based on the tuning of activation thresholds [109]. Mechanistically, the involvement of CD5 was thought to play a role in the modulation of FA [110]. The model we suggest is not new with regard to the fact that FA can be regulated/tuned. Rather, we propose that FA is constantly regulated for keeping FA stable, with the aim that T cells at different activation stages and with different affinity TCRs have similar FA, such that the T cells can respond in a similar fashion, rather than behave largely different and outcompete each other due to different FA.

We favor the hypothesis that FA is controlled by modulation of coreceptors and their signaling strength rather than modulation of individual (effector) functions because we think the former is capable to harmonize all functions of T cells for equal sensitivity to cognate antigen‐bearing cells. Modulation at the level of individual T‐cell functions would require paralleled and synchronized changes for the different functions (proliferation, migration, cytokine production, killing), which seems less likely since the different T‐cell functions are controlled by distinct transcriptional programs.

Since the mechanisms of coreceptor signaling and immune synapse functions are highly complex, many further studies are necessary to determine the accuracy and usefulness of our new model. It is designed to satisfy the conjecture that FA remains stable over time in vivo and is similar among the participating T cells, despite expressing TCRs with different affinities. We think that this only applies to those cells that have TCR affinities within a range that mediates optimal and equal function (plateau, Fig. 2C). The ones outside of a plateau may not significantly participate in the response and are therefore less likely to be relevant for protection.

## 10 Can one optimize vaccination strategies for better FA?

Many therapeutic propositions have been made for improving the FA. One of the key factors discussed has been the antigen dose used for vaccination [111]. For many years, it was understood that low antigen density led to the preferential recruitment of T cells with high FA, as they would outcompete the low avidity cells. Conversely, that a higher dose led to CD8 T cells with reduced FA [48, 112]. This dogma has persisted despite recent evidence that identifies further complexity and instances where high dose had no impact on the CD8 T cell's FA. Narayan et al. found that altering the amount of peptide when immunizing mice (for homologous prime/boost) had no impact on the FA of the CD8 T‐cell response [113]. In metastatic melanoma patients, it was shown that high vaccine doses induced higher frequencies of antigen‐specific CD8 T cells in the blood [22, 23]. Moreover, studying CD8 T‐cell responses over longer periods of time showed that repeated monthly vaccinations with high antigen dose in melanoma patients promoted Melan‐A‐specific CD8 T cells of high affinity and high FA [114].

While there is much evidence for an increased dose leading to increased magnitude of response [115, 116], there are currently no clear links between vaccine dose and FA of the primary response. Experiments in mice recently showed that low dose did in fact enhance FA; however, it was only observed for CD4 T cells and had no impact on the CD8 T cells [117]. Since the immune synapse differs between the CD4 and CD8 T cells, one cannot easily convey findings from one to the other [118]. Antigen dose is probably tightly associated with epitope density. One article found that increasing the epitope density on murine DCs increased the magnitude of the CD8 T‐cell response, but not the avidity of the primary response. However, it did impact on the avidity of recall responses [119]. Another study found that different epitope densities on murine DCs all lead to similarly high avidity T cells, showing independence to antigen density [120]. Overall, the data indicate that the immune system selects FA relatively independently of antigen dose or density. Since high antigen dose does not appear to hamper FA, we believe that one should no longer hesitate to use high antigen dose for vaccination.

Another tactic used for improving FA is the use of heterologous prime/boost vaccination, whereby one primes with one particular peptide, and follows the boost with another. Altered peptide ligands (APLs) were used, with one or more amino acid substitutions designed to improve MHC binding. Vaccination with such APLs can induce higher T‐cell frequencies [121, 122]. However, the popularity of APLs and their use for clinical cancer vaccine development has waned over the years, as it was discovered that vaccination with APLs had negative effects on the TCR repertoire and FA. This resulted in suboptimal T‐cell interaction with the original target antigen expressed by the tumor cells [123, 124]. It still remains to be determined whether or not in vivo immunization with optimized APLs can induce TCRs with the desired fine specificity [125].

Smart vaccine designs have been shown to improve T‐cell function. Vaccine adjuvants, such as TLR ligands promote inflammation and may improve FA indirectly, through improved cytokine production. The use of three different TLR ligands as a combination adjuvant induced qualitative changes in T‐cell responses needed for antiviral protection in mice [126]. In several other mouse studies, combinations of costimulatory molecules have been used to increase FA [127, 128]. There is also evidence in humans that some cytokines, such as IL‐12, may support T‐cell activation [129]. And incorporation of IL‐15 or expression of IL‐15 by a vaccine vector has been shown to select for murine T cells with higher FA [130]. Further research is needed to determine the mechanisms of how vaccine‐inducible inflammatory triggers and cytokines improve FA.

In contrast to immunotherapy with drugs or vaccines, adoptive T‐cell therapy provides an opportunity to impose a certain TCR affinity and consequent FA. High affinity TCRs can be selected or engineered to generate genetically modified T cells of enhanced FA (reviewed in Refs. [131, 132]). Adoptive transfer therapies require preconditioning of patients in order to deplete their own lymphocyte pool, allowing the transfused T cells to take over and dominate the immune response. Therefore, with this strategy one can establish high affinity/high FA cells that overrule the natural T‐cell population with its inherent FA. One possible target is the human tumor antigen NY‐ESO‐1, which is attractive because it is highly tumor specific and expressed in many cancers. Researchers achieved encouraging clinical results in patients with melanoma, sarcoma, and multiple myeloma. Patients were treated with T cells that had been engineered with NY‐ESO‐1‐specific TCRs designed for increased affinity as compared to naturally occurring TCRs [133, 134, 135]. However, adoptive transfer therapy with T cells expressing high affinity TCRs may potentially cause major adverse events due to the high capacity of destroying antigen‐bearing cells, particularly when the targeted antigens are also expressed by some healthy tissues [136]. Alternatively, future adoptive transfer therapies may be improved by engineering of coreceptors and related genes, based on enhanced understanding of their mechanisms and functions.

## 11 Concluding remarks

FA has a major impact on the success of immune defense. It may have superior importance over other correlates of CD8 T‐cell protection, because it defines the majority of them including the magnitude, the (multi‐)functionality, and the longevity of the CD8 T‐cell response. T cells with higher affinity TCRs are on average more powerful (but not beyond a certain maximal affinity) than those with lower affinity TCRs. However, natural polyclonal T‐cell responses include a range of TCR affinities that appear to develop a naturally defined FA, which usually does not depend on vaccine dose or boosting schedule, and which is remarkably stable. This stability is observed during the primary immune response. In contrast, the TCR clonotypic dominance and FA may change in the secondary response. Furthermore, most studies on long‐term CD8 T‐cell responses to persistent antigen showed that the FA remained stable over years.

Vaccines that modulate inflammatory parameters may improve FA, likely involving modulation of TCR affinity‐independent mechanisms. Alternatively, adoptive transfer of engineered T cells with carefully selected TCRs remains a promising option to establish optimal affinity T cells in patients with insufficient endogenous T‐cell responses. It is possible that our new model of constant FA regulation and these interpretations must be modified upon improved future knowledge of FA regulation, particularly regarding the dynamics of the immune synapse with its coreceptors and signaling mechanisms. Understanding how the synapse modifies T‐cell interactions and consequently the T‐cell functionality may give novel clues for improving immunotherapy.

## Conflict of interest

The authors declare no commercial or financial conflict of interest.

**Abbreviations**
APCs: antigen presenting cells
APLs: altered peptide ligands
FA: functional avidity
NTAmers: pMHC tetramers with Ni^2+^‐nitrilotriacetic acid‐His‐tag
PD‐1: programmed cell death protein‐1
pMHC: peptide‐MHC
SLAM: signaling lymphocytic activation molecule
SPR: surface plasmon resonance
